# Is age a determinant in cervical cancer screening in women aged 18 to 29?: An observational study

**DOI:** 10.1097/MD.0000000000042765

**Published:** 2025-06-13

**Authors:** Ayça K. Küçükyurt, Nil Atakul, Selma Atiye Kolcu, Ayşet Jane Özcan, Narmin Abdullazade

**Affiliations:** a Department of Obstetrics and Gynecology, İstanbul Training and Research Hospital, İstanbul, Turkey; b Department of Obstetrics and Gynecology, Altinbas University Faculty of Medicine, İstanbul, Turkey.

**Keywords:** cervical cancer, colposcopy, cytology, HPV, smear

## Abstract

This study aims to analyze the impact of aging on cervical cancer screening among women aged 20 to 29. Specifically, it examines the occurrence of abnormal histological results and Cervical Intraepithelial Neoplasia (CIN) (+) lesions within these age groups. We retrospectively analyzed women aged 18 to 29 (<30) who underwent cervical cancer screening through cytology between 2014 and 2024. A total of 842 women who visited the gynecology outpatient clinic were included, and their Pap smear, colposcopy, and loop electrosurgical excision procedure/conization results were reviewed. Patients were divided into 2 age groups (18–25 and 26–29) for comparative analysis. Among the 842 women who underwent cytological evaluation: 744 (88.4%) had normal results, 51 (6.1%) were diagnosed with atypical squamous cells of undetermined significance (ASC-US), 31 (3.7%) with low-grade squamous intraepithelial lesion (LSIL), 8 (1.0%) with atypical squamous cells – cannot exclude high-grade lesion (ASC-H), 4 (0.5%) with High-Grade Squamous Intraepithelial Lesion (HSIL), and 4 (0.5%) with Atypical Glandular Cells (AGC). HPV (Human Papillomavirus) testing was performed on 75 women, and 40 (53.3%) tested positive for HPV. Colposcopy was conducted on 53 women, revealing CIN + lesions in 38 cases, while conization confirmed CIN + lesions in 23 cases. Colposcopy results revealed the presence of CIN 1, CIN 2, and CIN 3 lesions in both age groups. However, CIN 3 lesions were observed less frequently in the > 26 age group. Similarly, conization results showed a lower incidence of CIN 3 lesions in women older than 26. Women aged 18 to 25 had a higher likelihood of CIN 3 diagnosis. These findings emphasize the importance of considering age in the diagnosis of CIN (+) lesions during cervical cancer screening.

## 1. Introduction

Cervical cancer is a significant health issue among women and poses a substantial global health burden. According to the World Health Organization, cervical cancer is one of the most common cancer types in women, ranking fourth among all female cancers. In 2022, an estimated 660,000 women were diagnosed with cervical cancer worldwide and about 350,000 women died from the disease..^[1,2]^

Cancer incidence and cancer-related mortality vary depending on economic development and lifestyle differences among countries. Approximately 90% of new cases and deaths occur in low and middle-income countries. In particular, cervical cancer ranks first in Eastern Africa with a high incidence rate of 40.1%.^[3]^

In Turkey, it is responsible for 5% to 10% of female cancers and ranks ninth.^[4]^

One of the most significant factors influencing cervical cancer is HPV (human papillomavirus) infection. Women infected with HPV have a 250- to 400-fold higher risk of developing cervical cancer. Additionally, several other factors contribute to an increased risk of cervical cancer, including: low socioeconomic status, smoking, engaging in sexual activity before the age of 18, having multiple sexual partners, giving birth multiple times, and having the first childbirth at an early age.^[5,6]^ The preinvasive stage of the cervix which is visible externally along with the slow progression of the disease, provides an opportunity for early screening and treatment.^[7]^

Regular screening tests especially the Pap smear test help detect precancerous (preinvasive) and cancerous cells at an early stage.^[8,9]^ Early detection allows for timely treatment playing a critical role in reducing cervical cancer-related mortality. However, there is no international consensus on the appropriate age to start screening. The American College of Obstetricians and Gynecologists and the American Cancer Society recommend: Primary HPV testing every 5 years for women aged 25 to 65. If primary HPV testing is unavailable screening can be done with co-testing (Pap smear + HPV test) every 5 years or Cytology (Pap smear alone) every 3 years. For women over 65 cervical cancer screening is not recommended if: They have no history of high-grade cervical intraepithelial neoplasia (CIN 2 or higher) or severe disease in the last 25 years and their screening results have been negative for the past 10 years.^[10,11]^ In Turkey it is recommended that the Pap smear test be performed every 5 year**s** for women aged 30 to 65.^[12]^

The Bethesda System is a method used for the classification and reporting of Pap smear results. Updated in 2001 this system provides a more accurate identification of cervical cancer and premalignant lesions.^[13]^

The classification includes:

Epithelial cell abnormalities

- Atypical squamous cells (ASC), further divided into:
- ASC-US (ASC of undetermined significance), and
- ASC-H (ASC – cannot exclude high-grade lesion).
- Low-grade squamous intraepithelial lesion (LSIL).
- High-grade squamous intraepithelial lesion (HSIL).
- Squamous cell carcinoma.

Glandular cell abnormalities

- Atypical glandular cell.
- Endocervical adenocarcinoma in situ.
- Adenocarcinoma.

Nearly all cervical cancers are associated with high-risk HPV infections. HPV types 16 and 18 are responsible for approximately 70% of all cervical cancers. Other high-risk HPV types 31, 33, 35, 39, 45, 51, 52, 56, 58, 68, 73, and 82.

Long-term HPV infection is believed to lead to cervical intraepithelial neoplasia (CIN) in a slow and sequential process. Progression from HPV-infected normal tissue can develop as follows: CIN 1, CIN 2, CIN 3 and ultimately, squamous cell carcinoma (CIS – carcinoma in situ). Current classification CIN 1 is referred to as LSIL, while CIN 2 and CIN 3 are referred to as HSIL.^[14]^

This study examines the impact of age on cervical cancer screening outcomes in women aged 18 to 29. Specifically, it analyzes age-related differences in HPV infection rates and CIN development, assessing the effectiveness of screening strategies in younger women. This research aims to evaluate the benefits of early screening and identify appropriate screening strategies for young women.

## 2. Materials and methods

Approval for this study was obtained from the Istanbul Training and Research Hospital Non-Interventional Clinical Research Ethics Committee with decision number 122 dated November 29, 2024. This retrospective study analyzed women aged 18 to 29 (<30) who underwent cervical cancer screening through cytology between 2014 and 2024. The study was conducted in accordance with the ethical standards outlined in the 2000 revision of the 1975 Declaration of Helsinki. A total of 1028 women who presented to the gynecology outpatient clinic were initially reviewed for their Pap smear, colposcopy, and loop electrosurgical excision procedure (LEEP)/conization results. After applying eligibility criteria, 842 women were included in the study. 183 patients were excluded due to missing results, and 3 pregnant patients were also excluded.

Patients were categorized into 2 age groups: 18 to 25 years, 26 to 29 years. When all patients are scanned, the patient group that meets the inclusion criteria is between the 2 most frequently mentioned age groups.

### 2.1. Exclusion criteria

- Patients with a history of CIN or gynecological cancer.
- Pregnant women.
- If a patient had multiple cytology results during the study period, the age at which abnormal cytology was observed was recorded. If all cytology results were normal, the mean age across all screenings was considered.
- Patients with endocervical/transformation zone-only cytology results were excluded.

Data collection demographic and clinical data were collected, including:

- Marital status.
- Obstetric and gynecological history (pregnancy, number of births, number of miscarriages).
- Use of contraceptive methods.
- Smoking status.
- Cytology results.
- HPV test results (if available).
- LEEP/conization results.

Patients with CIN lesions who did not undergo colposcopy were excluded from the study.

### 2.2. Cytology analysis

All samples were analyzed using liquid-based cytology. Normal cytology was defined as negative for intraepithelial lesion or malignancy (NILM). Cytology results were classified according to the Bethesda System, including ASC of undetermined significance (ASC-US), ASC – cannot exclude high-grade lesion (ASC-H), Low-grade squamous intraepithelial lesions (LSIL), High-grade squamous intraepithelial lesions (HSIL), Atypical glandular cells (AGC), Adenocarcinoma in situ and invasive cancer.^[15]^

### 2.3. Management of abnormal cytology results

The management of abnormal cytology results was conducted according to the American Society for Colposcopy and Cervical Pathology guidelines. Women with ASC-US, LSIL, HSIL, ASC-H, AGC, or high-risk HPV positivity were referred for colposcopy. Additionally, colposcopy was performed in cases of postcoital bleeding, visible cervical lesions, or condyloma acuminatum.

### 2.4. Colposcopy procedure

During the colposcopy, a 3% acetic acid solution was applied to the cervix, and after 30 to 90 seconds, the cervical cells were dried. This solution causes squamous cells with large or dense nuclei (e.g., metaplastic, dysplastic, or HPV-infected cells) to appear white due to their light-reflecting properties. This acetowhite effect is an important indicator for precancerous lesions and HPV infections. Following these staining techniques, colposcopic imaging was performed, and biopsy results were classified as: Normal (no CIN lesion), CIN 1, CIN 2, CIN 3, invasive cancer.^[16]^

### 2.5. Histological correlation

- CIN 1 lesions were classified as histologically confirmed LSIL.
- CIN 2 and CIN 3 lesions were classified as histologically confirmed HSIL.

### 2.6. Follow-up strategy

- Patients with ASC-H, AGC, or HSIL underwent colposcopy.
- If colposcopic findings were negative, cytology was repeated after 12 months.
- Patients with negative cytology at follow-up were documented accordingly.^[16–18]^

### 2.7. Statistical methods

Descriptive statistics for the data included mean, standard deviation, median, minimum, maximum, frequency, and percentage values. The distribution of variables was assessed using the Kolmogorov-Smirnov and Shapiro–Wilk tests. For non-normally distributed continuous independent variables, the Mann–Whitney *U* test was used. For categorical independent variables, the Chi-square test was applied. If Chi-square test assumptions were not met, the Fisher exact test was used. All statistical analyses were conducted using SPSS version 27.0.

## 3. Results

A total of 1028 women aged 18 to 29 were screened for Pap smear, colposcopy, and LEEP/conization results. Based on the screening criteria, 842 patients were included in the study (Table 1). 183 patients were excluded due to missing results, and 3 patients were excluded due to pregnancy. Among them, 718 were married, and 124 were in nonmarital relationships. Among the participants, 25 were smokers, 298 had given birth, and 218 were using contraception. Regarding contraceptive methods, it was observed that 74 women used oral contraceptives (OCPs), 49 had an intrauterine device (IUD) inserted, 58 used condoms, 5 received injectable contraception, and 32 preferred the withdrawal method (coitus interruptus).

**Table 1:** Demographic characteristics (aged 18–29).

		Min–max	Median	Mean ± sd, n-%	
Age		18.0–29.0	26.0	25.2 ± 2.8	
Marital status	Married			718	85.3%
	sex/active			124	14.7
Smoking status	(−)			817	97.0
	(+)			25	3.0
Parity	(−)			544	64.6
	(+)			298	35.4
Contraceptive	(−)			624	74.1
	(+)			218	25.9
Oral contraceptive pills				74	33.9
IUD				49	22.5
CI				32	14.7
Condom				58	26.6
Inj.				5	2.3

CI = coitus interruptus, Inj. = injectable contraception, IUD *=* intrauterine device, sd = standard deviation.

Among the 842 women who underwent cytological examination, 744 (88.4%) had normal results, while 51 (6.1%) were diagnosed with ASC-US, 31 (3.7%) with LSIL, 8 (1.0%) with ASC-H, 4 (0.5%) with HSIL, and 4 (0.5%) with AGC. HPV testing was performed on 75 women, and 40 (53.3%) tested positive for HPV. Colposcopy was conducted in 53 women, revealing CIN + lesions in 38 cases, while conization confirmed CIN + lesions in 23 cases (Table 2).

**Table 2:** Clinical parameters in patients aged 18 to 29 with normal cytology/colposcopy or histologically diagnosed CIN (+) lesions.

		n	%
Smear	Normal	744	88.4
	Anomaly	98	11.6
AS-CUS		51	52.1
LSIL		31	31.7
HSIL		8	8.1
ASC-H		4	4.05
AGC		4	4.05
Repeat test in cases of insufficient smear	Normal	21	100
HPV	(−)	767	91.1
	(+)	75	8.9
Negative		35	46.7
High-risk positive		27	36.0
Low-risk Positive		13	17.3
Colposcopy	(−)	789	92.9
	(+)	53	6.3
CIN 1		11	20.7
CIN 2		13	24.5
CIN 3		14	26.4
* Ch. Cervicitis*		15	28.4
Conization	(−)	815	96.7
	(+)	27	3.3.
CIN 1		7	17.9
CIN 2		7	17.9
CIN 3		9	35.9
* Ch. Cervicitis*		4	38.5

AGC = atypical glandular cells, ASC-H = atypical squamous cells – cannot exclude high-grade lesion, ASC-US = atypical squamous cells of undetermined significance, *Ch. Cervicitis =* chronic cervicitis, CIN = cervical intraepithelial neoplasia, HPV = human papillomavirus, HSIL = high-grade squamous intraepithelial lesion, LSIL = low-grade squamous intraepithelial lesion.

In Table 3, the results of the < 26 and ≥ 26 age groups were evaluated. The proportion of married individuals was significantly higher in the ≥ 26 age group compared to the < 26 age group (*P* < .05). However, no significant difference was observed between the 2 groups regarding smoking rates (*P* > .05). The parity number was significantly higher in the ≥ 26 age group than in the < 26 age group (*P* < .05). There was no significant difference in contraception usage rates between the 2 groups (*P* > .05). Additionally, there were no significant differences between the < 26 and ≥ 26 groups in terms of Pap smear results, HPV positivity, colposcopy, and conization rates (*P* > .05) (Table 3).

**Table 3:** Clinical parameters in patients aged < 26 and ≥ 26 with normal cytology/colposcopy or histologically diagnosed CIN (+) lesions based on demographic, cytological, colposcopy, and LEEP findings.

		Age < 26 (n = 420)		Age ≥ 26 (n = 422)		*P*	
		Average ± ss/n-%	Median	Average ± ss/n-%	Median		
Marital status	Married	326	77.6%	392	92.9%	**.000**	χ^2^
	Sexually active	94	22.4%	30	7.1%		
Smoking status	(−)	406	96.7%	411	97.4%	.534	χ^2^
	(+)	14	3.3%	11	2.6%		
Parity	(−)	320	76.2%	224	53.1%	**.000**	χ^2^
	(+)	100	23.8%	198	46.9%		
Contraceptive	(−)	315	75.0%	309	73.2%	.556	χ^2^
	(+)	105	25.0%	113	26.8%		
Smear Result	Normal	370	88.1%	374	88.6%	.810	χ^2^
	Anomaly	50	11.9%	48	11.4%		
HPV	(−)	381	90.7%	386	91.5%	.701	χ^2^
	(+)	39	9.3%	36	8.5%		
Colposcopy	(−)	395	94.0%	394	93.3%	.690	χ^2^
	(+)	25	6.0%	28	6.7%		
Conization	(−)	402	95.7%	413	97.8%	.628	χ^2^

Statistically significant values (P < .001) are indicated in bold.

CIN = cervical intraepithelial neoplasia, HPV = human papillomavirus, LEEP = loop electrosurgical excision procedure.

In Table 4, the distribution of HPV test and colposcopy results by age groups was analyzed. In the < 26 age group (n = 420), 37 women underwent HPV testing, revealing high-risk HPV in 14 (37.83%), low-risk HPV in 14 (37.83%), and negative results in 9 (24.32%). HPV testing was not performed in 383 women. Among 25 women who underwent colposcopy, 4 (0.95%) had CIN 1, 6 (1.42%) had CIN 2, and 9 (2.14%) had CIN 3. Among 14 women who underwent conization, 4 (0.95%) had CIN 1, 4 (0.95%) had CIN 2, and 5 (1.19%) had CIN 3. In the ≥ 26 age group (n = 422), 38 women underwent HPV testing, with high-risk HPV detected in 5 (13.16%), low-risk HPV in 15 (39.47%), and negative results in 18 (47.37%). HPV testing was not performed in 384 women. Among 28 women who underwent colposcopy, 7 (1.65%) had CIN 1, 7 (1.65%) had CIN 2, and 5 (1.18%) had CIN 3. Among 13 women who underwent conization, 3 (0.71%) had CIN 1, 3 (0.71%) had CIN 2, and 4 (0.94%) had CIN 3. The proportion of HPV-tested patients was 8.8% in the < 26 group and 9.0% in the ≥ 26 group, with a high rate of untested patients in both groups. Colposcopy findings showed CIN 1, CIN 2, and CIN 3 lesions in both groups, but CIN 3 lesions were less frequent in the ≥ 26 group. Similarly, conization results indicated a lower incidence of CIN 3 in the ≥ 26 group.

**Table 4:** Clinical parameters in patients aged < 26 and ≥ 26 with normal cytological/colposcopic findings or histologically diagnosed CIN (+) lesions.

	Age < 26	n = 420	%	Age ≥ 26	n = 422	%
Smear	ASC-US	18	4.28	ASC-US	14	3.31
	LSIL	15	3.57	LSIL	15	3.55
	HSIL	3	0.71	HSIL	4	0.94
	ASC-H	3	0.71	ASC-H	1	0.23
	AGC	0	0	AGC	1	0.23
Hpv	no HPV	383	91.5	no HPV	384	91.5
	Negative	14	3.35	Negative		4.26
	High risk	14	3.35	High risk	18	1.18
	Positive			Positive	5	
	Low-risk positive	9	1.8	Low-risk positive	15	3.55
Colposcopy	CIN 1	4	0.95	CIN 1	7	1.65
	CIN 2	6	1.42	CIN 2	7	1.65
	CIN 3	9	2.14	CIN 3	5	1.18
	Chr cervicitis	6	1.42	Chr cervicitis	9	2.13
Conization	CIN 1	4	0.95	CIN 1	3	0.71
	CIN 2	4	0.95	CIN 2	3	0.71
	CIN 3	5	1.19	CIN 3	4	0.94
	Chr cervicitis	1	0.23	Chr cervicitis	3	0.71

AGC = atypical glandular cells, ASC-H = atypical squamous cells – cannot exclude high-grade lesion, ASC-US = atypical squamous cells of undetermined significance, *Ch. Cervicitis =* chronic cervicitis, CIN = cervical intraepithelial neoplasia, HPV = human papillomavirus, HSIL = high-grade squamous intraepithelial lesion, LSIL = low-grade squamous intraepithelial lesion.

## 4. Discussion

In our study, we analyzed the impact of age on cervical cancer screening by examining the demographic characteristics, HPV status, cytology, and histology results in women aged 18 to 29. According to our findings, 11.6% of women had abnormal cytology results, while 8.9% of women in the 18 to 29 age group tested positive for HPV. Additionally, we investigated the effect of age on the development of CIN (+) lesions in this population. Colposcopy results revealed the presence of CIN 1, CIN 2, and CIN 3 lesions in both age groups; however, CIN 3 lesions were less frequently observed in the > 26 age group. Similarly, conization results showed a lower incidence of CIN 3 in women older than 26. When reviewing the literature, a study conducted by Tao et al (2021) reported that the ASC-US detection rate was 6.4% in women < 20 years old, 3.6% in women aged 20 to 29, and increased to 5.5% in women aged 50 to 59. Among women aged ≥ 70 years, the ASC-US reporting rate was 5.1%, showing an increase with age. The study also found that the highest high-risk HPV positivity rate was in women < 20 years old (69.4%), whereas the lowest rate was in women ≥ 70 years old. Contrary to Tao et al findings, our study showed that women < 26 years old had higher ASC-US and high-risk HPV positivity rates.^[19]^ Campaner et al (2023) reported that low-grade lesions peak in incidence before the age of 30, while the rates of ASC-US and AGC increase in older women. In our study, AGC was not observed in the < 26 age group, whereas it was detected at a rate of 0.23% in the > 26 age group. Additionally, the ASC-US rate was 4.28% in the < 26 age group, which was higher than in the > 26 age group.^[20]^

It has been reported that HPV 16/18 and HSIL lesions are twice as common in women under 30, whereas in older women, other HPV types are more frequently detected. However, since our study exclusively examined women aged 18 to 29, we were unable to directly address this finding. In our study, we observed that high-risk HPV infections had a higher prevalence in women aged 18 to 25, while HPV positivity was lower in women aged 26 to 29.^[21]^

Tanik et al (2025) reported that the high-risk HPV positivity rate in the 18 to 24 age group was 28.7%, and that individuals with HPV positivity had a 32.59% higher risk of LSIL and HSIL cytology compared to HPV-negative individuals. In our study, although a high-risk HPV positivity rate was observed in the 18 to 25 age group, no significant difference was found in LSIL and HSIL rates.^[22]^

Chen et al (2015) reported that ASC-H was observed in 42% of women under 30, with HR-HPV positivity in 95.2% of women under 20. They also found that the detection rate of CIN2 + in ASC-H cases was 51.2% in women under 20. In our study, ASC-H was detected in 0.71% of women in the < 26 age group and 0.23% in the > 26 age group.^[23]^

Zhong et al (2020) categorized AGC into AGC not otherwise specified (AGC-NOS) and AGC favoring neoplasia (AGC-FN). Their study reported that 15.6% of AGC cases occurred in women under 31 and over 60 years old, with AGC-FN detected in 21.8% of cases. Since our study focused on women aged 18 to 29, a direct comparison is limited. However, in the 26 to 29 age group, we identified 1 case of AGC.^[24]^

Xiao et al (2023) reported that AGC was more common in women aged 40 and above compared to those under 40. They also found that in AGC patients, the likelihood of an abnormal origin from glandular epithelium was higher than from squamous epithelium. Similarly, in our study, no AGC cases were observed in the 18 to 25 age group, while in the 26 to 29 age group, AGC was detected at a rate of 0.23%.^[25]^

Sakai et al (2022) divided patients into 4 age groups and examined the distribution of high-grade HPV positivity by cytology in each group. They reported that in the 18 to 29 age group, the most common combination was HSIL and HPV 16/18 positivity, with 73% of CIN2 + cases consisting of HSIL and other HPV-positive cases. Similarly, in our study, the high-risk HPV positivity rate in the < 26 age group was 3.35%, while the CIN3 + rate was 2.14%.^[26]^

Giannella et al (2021) reported that in women under 30, the prevalence of non-HPV 16/18 types was lower, but the proportion of CIN 3 lesions and non-HPV 16/18 infections increased with age. In contrast to their findings, our study showed that CIN 3 lesions were more frequent in the < 26 age group compared to the > 26 age group, based on both colposcopy and conization results.^[27]^

Onuki et al (2009) reported that the highest rates of invasive cervical cancer (90.0%) and CIN2 to 3 (53.9%) were observed in the 20 to 29 age group, with these rates decreasing with age, reaching the lowest levels in women over 60. In our study, CIN 1 and CIN 2 lesions were more frequently observed in colposcopy results in the > 26 age group compared to the < 26 age group.^[28]^

Tuncer et al (2020) analyzed smear, HPV, and colposcopy results in women aged 20 to 29 and found that women aged 25 to 29 had a higher likelihood of CIN + compared to those aged 20 to 24. Contrary to their findings, our study showed that the likelihood of CIN + was higher in the 18 to 25 age group compared to the 26 to 29 age group. However, the overall CIN + rate was the same in both the < 26 and > 26 age groups.^[29]^

Qin et al (2020) reported that between 2011 and 2017, more than half of bimanual pelvic examinations and nearly 3-quarters of Pap tests performed in young women aged 15 to 20 were potentially unnecessary.^[30]^

Castanon et al (2020) conducted a study in the United Kingdom to observe cervical cancer cases detected through screening and document survival rates. They reported that in stage IB cervical cancer, the survival rate was worse in women diagnosed at ages 20 to 24 compared to those diagnosed at ages 25 to 29. Unlike their study, our research focused on colposcopy and conization results in patients with abnormal smear findings and high-risk HPV positivity. In the < 26 age group, we observed that CIN 3 lesions were present in 2.14% of cases, while high-risk HPV positivity was 3.35%, indicating a higher prevalence in this age group.^[31]^

Torres et al (2020) reported that the youngest age group (18–24 years) had the highest prevalence of any type of HPV infection, at 26.7%. In our study, no significant difference was found between the 18 to 25 and 26 to 29 age groups regarding overall HPV prevalence. However, we observed that high-risk HPV positivity was higher in the < 26 age group.^[32]^

Huică et al (2019) reported that the highest percentage of high-risk HPV infections was found in the 30 to 39 age group and those under 30. In contrast, our study found that high-risk HPV infections were more prevalent in the 18 to 25 age group, whereas low-risk HPV infections were more common in the 26 to 29 age group.^[33]^

Pruski et al (2012) investigated the regression and progression frequency of mild cervical neoplasia (LSIL) in 111 women positive for HPV DNA types with high oncogenic potential. Cervical cytology was performed every 3 months and colposcopy every 6 months for 1 year, and colposcopy was performed at the end of 12 months and biopsies were taken. According to the results, regression of LSIL was more common in women under 26 years of age than in older women; Progression to HSIL was more common in women over 36 years of age. In our study, LSIL was seen in the same number in both groups aged < 26 years. HSIL was seen more in > 26 years. However, CIN 3 lesions were seen less frequently in the > 26 Ibid group. Conization results also show lower CIN 3 rates in the > 26 age group.^[34]^

In the study by Kedzia et al (2010), as a result of genotyping performed on 126 women with HPV DNA positivity and diagnosed with CIN 1, the most common oncogenic HPV types were determined as HPV 16, 33, 18, 31 and 45, respectively. In our study, HPV positivity (types 16 and 18) showed a higher risk in the age group < 26 compared to the age group > 26.^[35]^

## 5. Conclusions

Cervical cancer screening plays a crucial role in women aged 18 to 29, and age has significant effects on HPV infection, as well as cytological and histological outcomes. In our study, HPV positivity and abnormal cytological results were observed at a notable rate among women in this age group. When evaluating the impact of age on the development of CIN (+) lesions, we found that CIN 3 lesions were less frequent in the > 26 age group. Compared to previous studies in the literature, our findings exhibit some differences, particularly in the higher prevalence of high-risk HPV infections in the 18 to 25 age group. Additionally, in the 26 to 29 age group, HPV positivity rates were lower, and CIN 3 lesions were detected less frequently. The limitation of this study is its retrospective design. In conclusion, our study highlights the need for an age-based approach in cervical cancer screening and emphasizes the importance of regular screening and follow-up in women aged 18 to 29. These findings suggest that future screening protocols should be tailored more specifically to age-related risk factors.

## Acknowledgments

We would like to thank statistician Ertan Koç for his statistical evaluation of the manuscript.

## Author contributions

**Conceptualization:** Ayça K. Küçükyurt, Nil Atakul.

**Data curation:** Nil Atakul, Narmin Abdullazade.

**Investigation:** Narmin Abdullazade.

**Methodology:** Ayça K. Küçükyurt, Nil Atakul.

**Project administration:** Nil Atakul, Selma Atiye Kolcu.

**Resources:** Ayça K. Küçükyurt, Selma Atiye Kolcu, Ayşet Jane Özcan.

**Supervision:** Ayşet Jane Özcan.

**Validation:** Ayça K. Küçükyurt.

**Writing –review & editing:** Ayça K. Küçükyurt, Nil Atakul, Selma Atiye Kolcu, Ayşet Jane Özcan, Narmin Abdullazade.
